# Overexpression of proteasomal activator PA28α serves as a prognostic factor in oral squamous cell carcinoma

**DOI:** 10.1186/s13046-016-0309-z

**Published:** 2016-02-19

**Authors:** Xiaodong Feng, Yuchen Jiang, Liang Xie, Lu Jiang, Jing Li, Chongkui Sun, Hao Xu, Ruinan Wang, Min Zhou, Yu Zhou, Hongxia Dan, Zhiyong Wang, Ning Ji, Peng Deng, Ga Liao, Ning Geng, Yun Wang, Dunfang Zhang, Yunfeng Lin, Ling Ye, Xinhua Liang, Longjiang Li, Gang Luo, Mingye Feng, Juan Fang, Xin Zeng, Zhi Wang, Qianming Chen

**Affiliations:** State Key Laboratory of Oral Diseases, West China College of Stomatology, Sichuan University, No. 14, Sec.3, Renminnan Road, Chengdu, Sichuan 610041 China; State Key Laboratory of Biotherapy and Cancer Center, West China Hospital, West China Medical School, Sichuan University, Chengdu, China; Guangdong Provincial Key Laboratory of Oral Diseases, Stomatological Hospital, Guanghua School of Stomatology, Sun Yat-Sen University, Guangzhou, China; Key Laboratory of Oral Medicine, Guangzhou Institute of Oral Disease, Stomatology Hospital of Guangzhou Medical University, Guangzhou, China

**Keywords:** PA28α, OSCC, Prognosis, Recurrence, Overall survival

## Abstract

**Background:**

Despite recent advances in oral squamous cell carcinoma (OSCC) diagnosis and therapy, disease recurrence remains common and is strongly associated with mortality. It is therefore critical to identify new targets for both treatment and diagnostic purposes. We aimed at investigating the role of PA28α, a proteasomal activator in OSCC.

**Methods:**

The expression of PA28α was examined in a panel of OSCC cell lines and tissues, associated with oncomine analysis. In a large OSCC patient cohort, the prognostic value of PA28α expression was evaluated. Primary clinical end points were recurrence-free and overall survival rate. Functional involvement of PA28α in OSCC was examined in both in vitro and in vivo models upon specific siRNA knockdown.

**Results:**

PA28α was found to be overexpressed in OSCC cell lines and tumor tissues. High expression of PA28α was significantly associated with recurrence and poorer overall survival. Specific knockdown of PA28α inhibited OSCC cell proliferation, migration, invasion in vitro and reduced the growth of OSCC xenografts in vivo. Multivariate Cox regression analyses revealed PA28α as independent prognostic predictors.

**Conclusions:**

These results suggest that PA28α is involved in OSCC oncogenesis and may serve as a potential prognostic factor.

**Electronic supplementary material:**

The online version of this article (doi:10.1186/s13046-016-0309-z) contains supplementary material, which is available to authorized users.

## Background

Oral squamous cell carcinoma (OSCC) represents a major subset of head and neck cancer, which is the sixth most common cancer worldwide [1]. Data from the Oral Cancer Foundation indicates that there are more than 300,000 new cases of OSCC each year worldwide. Despite recent advances in diagnosis and therapy, disease recurrence remains common and is strongly associated with mortality [2, 3]. It is therefore critical to identify new targets for both treatment and diagnostic purposes.

Proteasomes are essential compartmentalized proteases for the viability of all eukaryotes and archaea. Proteasomal degradation is critically dependent on proteasome activators, which bind to the end of the 20S core particle (CP) for reposition of its gating residues and allow access to doomed substrates [4]. The 11S activators are a family of small proteins that form heptameric rings and enhance peptide degradation upon binding to 20S CPs without ATP. There are three 11S activator isoforms: PA28-α, -β, and -γ. PA28γ forms a homoheptamer, whereas PA28α and PA28β subunits form a heteroheptamer [5]. PA28γ can promote the degradation of multiple proteins including p53 and MDM2 in an ubiquitin- and ATP-independent manner. Most recent study showed that PA28γ is critical for skin carcinogenesis by modulating the Wnt/b-catenin pathway [6–8]. A multitude of studies have demonstrated that PA28-α/β could alter the peptidic products of proteasomal degradation to influence major histocompatibility complex class I antigen presentation. PA28α^–/–/^β^–/–^ mice showed apparently normal immune responses against influenza A virus infection, while completely lost the ability to process a melanoma antigen TRP2-derived peptide. Hence, PA28α/β is not a prerequisite for antigen presentation in general, but plays an essential role for the processing of certain antigens [9, 10]. More recently, upregulation of PA28α has been found to associate with several different cancers, including ovarian cancer and prostate cancer [11, 12]. However, its precise biological roles in tumorigenesis remain unclear.

In our previous study, the up-regulation of PA28α was detacted in OSCC cells and a small number of clinical samples [13, 14]. In this study, the expression level of PA28α was verified and its prognostic value was assessed in a large OSCC patient cohort. RNAi-mediated gene silencing was used to investigate the contribution of PA28α to OSCC cell proliferation, apoptosis, migration and invasion in vitro. Moreover, OSCC cells with reduced PA28α expression were inoculated into mice to assess the role of PA28α on tumor formation and growth in vivo.

## Methods

### Patients and tumor samples

The cohort comprised OSCC from 98 patients, who were treated with primary surgical resection with curative intent at West China Stomatological Hospital, Sichuan University (Chengdu, China) during the period of March 2002 to June 2009. The clinical and pathologic features of all patients were shown in Additional file 1: Table S1. All patients were examined routinely every 3 months during the first year of follow-up, then every 6 months for 4 years, and once a year thereafter. The mean post-operative follow-up period was 84 months. For immunohistochemical examination, tissues were fixed in 10 % buffered formalin and embedded in paraffin. The specimens were examined histologically after staining with H&E staining, and the clinicopathologic staging was determined according to the TNM classification system of the International Union against Cancer by West China Stomatological Hospital, Sichuan University (Chengdu, China). All tissue specimens used for staining were primary diagnostic pre-treatment samples. All of the patients or their relatives were given informed consents for the use of their tissues in the experimental procedures. The project was approved by the Scientific and Ethical Committee of Sichuan University, China.

### Cell lines and reagents

CAL27 and HSC3 Cells were cultured in DMEM (Gibco, Grand Island, NY, USA) supplemented with 10 % fetal bovine serum (FBS) (Gibco, Grand Island, NY, USA) and 100 units/ml penicillin, 100 ng/ml streptomycin. All cells were cultured in T75 tissue culture flasks under a humidified condition at 37 °C in 5 % CO2. Three siRNAs (siRNA#1, siRNA#2 and siRNA#3) (Ribobio, Guangzhou, China), were designed against Pa28α (GenBank accession No. NM_176783). One scrambled siRNA (si-Control) (Ribobio, Guangzhou, China), exhibiting no significant sequence similarity to human, mouse or rat gene sequence, was used as a negative control. Transfection of siRNA was performed with lipofectamine TM 2000 (Invitrogen, Grand Island, NY, USA) according to the manufacturer’s protocol.

### Q-PCR

Total RNA of each sample was extracted using Trizol reagent (Invitrogen, Grand Island, NY, USA). The reverse transcription was performed with the Reverse Transcription System (Promega, Madison, WI, USA). The primers used in the Q-PCR reaction were:

PA28α (Forward: 5′ –GCGCTTGAAGCCTGAGATCA-3′,

Reverse: 5′-CCTTCTCCTGGACAGCCACT-3′),

Beta-actin (Forward: 5′-TTAGTTGCGTTACACCCTTTC-3′,

Reverse 5′-CTGTCACCTTCACCGTTCC-3′).

Q-PCR was performed using the AVI 7300 system (ABI, Grand Island, NY, USA).

### MTT assay

MTT assay was used to determine the viability of cells upon treatment. A total of 3 × 103 HSC3 cells or CAL27 cells were seeded per well of a 96-well plate in triplicate. The cell viability was determined at 24, 48, 72 and 96 hours upon treatment using the MTT assay. Briefly, 20ul of MTT (Sigma, St. Louis, MO, USA) solution [5 mg/ml in phosphate buffered saline (PBS)] was added to each well and incubated for 4 hours. Then 100 μl DMSO was added into each well for solubilization of the formazan. Optical absorbance of the reaction was measured spectrophotometrically at 570 nm with an ELISA plate reader (Genios TECAN, Männedorf, Schweiz).

### Edu incorporation assay

Edu incorporation assay was used to determine the cell proliferation. At 24 hours post transfection with siRNAs, 2.5 × 10^4^ Cal27 and HSC3 cells were seeded per confocal dish in triplicate, after 12 hours incubated with DMEM supplemented with 50 uM Edu(RiboBio,China) for 2 hours. Cells were then washed with PBS twice, followed by 4 % paraformaldehyde fixation and incubation with glycine 2 mg/ml, washed with PBS three times, and permeabilized with PBS containing 0.5 % triton X-100. After washing with PBS three times, cells were incubated with Apollo staining solution for 30 min, washed with PBS twice, followed by 5 min incubation with DAPI. Photographs of the cells were captured with a laser scanning confocal microscopy.

### Colony formation assay

For plate colony assay, at 24 hours post treatment with siRNAs, 6 × 10^2^ HSC3 cells or CAL27 cells were reseeded into each well of 6-well plates. After culturing for 2 weeks, cells were stained with Crystal Violet (Sigma, St. Louis, MO, USA).

### TUNEL assay

Apoptosis was measured using the TUNEL Apoptosis Detection Kit (Upstate Inc. Madison, WI, USA). Cells were cultured on chamber slides overnight, treated with siRNA as indicated above. After treatment, cells were washed with PBS and fixed with 4 % methanol-free formaldehyde solution in PBS for 5 mins at room temperature. Staining was done according to the manufacturer's instructions. Fluorescence was visualized with Olympus BX60 microscope (Olympus Optical Co. Tokyo, Japan).

### Wound healing assay

Cells were seeded onto a 6-well plate at a density of 1 × 105 per well in growth medium overnight, and treated with siRNA as indicated above. After 24 hours, a single scratch wound was created using a micropipette tip. Cells were washed with PBS to remove cell debris, supplemented with assay medium, and monitored. Images were captured with a phase-contrast microscopy (×20) at 0, 24, and 48 hours.

### Cell transwell migration and invasion assays

The migration capability of cells through filters was assayed using a Boyden transwell chamber (with 8 μm pore size). At 24 hours after the treatment with siRNAs, the cells were seeded in to the upper chamber. The bottom chamber included medium (0.5 ml) containing 20 % FBS. After incubation for 24 hours or 10 hours, cells were stained with Crystal Violet (Sigma, St. Louis, MO, USA). Non-migrated cells on the upper surface of the filters were gently removed and cells migrated to the bottom side of the filter were counted under a light microscope. For the invasion assay, Matrigel (Collaborative Biomedical Products, Bedford, MA, USA) was diluted to 25 mg/50 ml with cold filtered medium without FBS, and applied to the 8 μm pore size polycarbonate membrane filters of the Boyden chamber. The Boyden chamber with Matrigel was used for the invasion assay. After incubating cells for 48 h, invaded cells were stained and counted the same way as above.

### Western blot analysis

Cell lysates were denatured in sample buffer containing SDS, and equal amounts of protein were separated on 8–15 % SDS-polyacrylamide gels and transferred to nitrocellulose membranes (Millipore, Bedford, MA, USA). After blocking with 5 % non-fat dry milk, the membranes were incubated overnight at 4 °C with the primary antibodies, Anti-PA28α (Abcam, Cambridge, MA, USA) and anti-β-actin (Cell Signaling Technology, Danvers, MA, USA), as indicated. In the subsequent day, the membranes were incubated with the appropriate horseradish peroxidase-conjugated secondary antibodies and subjected to chemiluminescence.

### Immunohistochemistry

Tissue sections were cut at 4um thickness, deparaffinized, rehydrated. Heat-based antigen retrieval was performed prior to incubation with the mouse monoclonal anti-human PA28α(at 1:200 dilution; Abcam, Cambridge, MA, USA) at 4 °C overnight. Bound antibodies were visualized using an Envision Kit (DAKO Corporation, USA) according to the manufacturer's instruction (DAKO Corporation, USA). After the expected stain intensity was developed, sections were lightly counterstained with hematoxylin. Sections treated without primary antibodies were used as negative controls.

### In vivo tumor formation assay

All animal studies were conducted following the U.S. Public Health Service policy on humane care and use of laboratory animals. Fifteen 4 to 8-week-old female BALB/c-nu/nu nude mice were purchased from the Animal Center of Beijing HFK Bio-Technology.co., LTD (Animal Center of Beijing HFK Bio-Technology.co., LTD, Beijing, China) and randomly divided into 2 groups with 5 mice each. 1.5 × 10^6^ viable HSC3 cells, which had been transfected with 2’ OMe and 3’ Chol modified siRNA-PA28α and siRNA-Control (Ribobio, Guangzhou, China), were harvested by trypsinization 12 hours after transfection, washed twice with 1 × PBS and resuspended in 100 μl of DMEM without FBS and antibiotics. Cells were then injected subcutaneously into the right flank site of each mouse (siRNA-PA28α, n = 5 and siRNA-Control, n = 5). Samples of the injected cells were also collected for the validation of PA28α knockdown at the time of injection. The mice were kept in a pathogen-free environment, and tumor size was measured by caliper every 6 days for 24 days. The tumor volume was calculated by the following formula: tumor volume = 1/2 × longer diameter × shorter diameter^2^. All the mice were sacrificed when the average tumor volume of the siRNA-Control group reached 350 mm3. Immunohistochemical analysis of PCNA, CD34 and TUNEL assay were performed in all tumors slides.

### Statistical analysis

We made use of mean ± standard deviation($\overline{x}\pm \mathrm{s}$)or quartile (*Q*_1_, *M* and *Q*_3_) to describe numerical variables, and percentage to describe categorical variables among our study population. Recurrence (recurrence verses (vs.) non-recurrence) and death (death vs. survival) were taken as evaluation indicator for clinical outcome of the patients and Kaplan-Meier method was utilized to calculate the cumulative non-recurrence probability and survival probability and draw the survival curves for our follow-up data. We used the Cox Hazard Model to filter the potential risk factors for recurrence and death comprehensively. The potential factors we selected included age, gender, employment, education, residence, marriage, smoking, drinking, alpha domain, alpha intensity, differentiation, T-stage, lymphatic metastasis, clinical stage, surgery, radiotherapy and chemotherapy. Univariate analysis was first utilized to evaluate all potential factors one by one. In order to obtain reasonable factors, we then selected these variables with *P* value less than 0.20 to enter multivariate Cox regression analysis. A *P* value of less than 0.05 was considered to indicate statistical significance, except for multivariate Cox regression, where a level of 0.10 was used for the exploratory study. Except the clinical data, all other data was performed using GraphPad Prism version 5 for Windows (GraphPad Software, San Diego, CA, USA). The data were analyzed by ANOVA test or t-test (* p < 0.05, ** p < 0.01, *** p < 0.001).

## Results

### PA28α expression pattern in clinical cohort

**Fig. 1:**
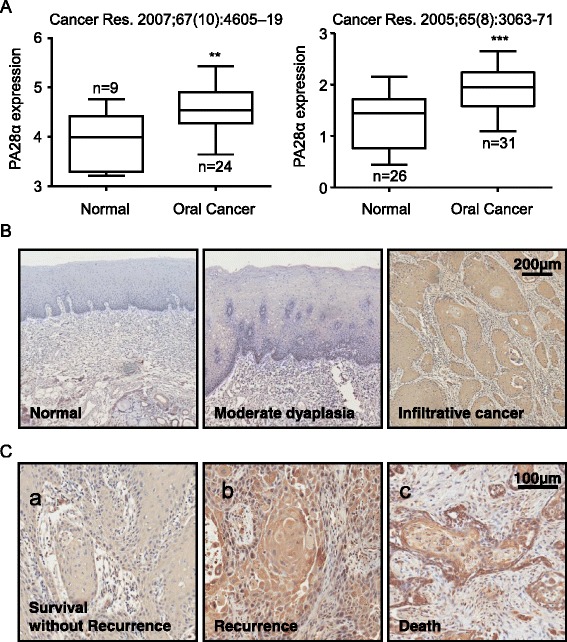
Expression of PA28α related to carcinogenesis and prognosis in OSCC tissues. **a** Oncomine analysis of PA28α mRNA abundance in human primary OSCCs compared with human normal oral tissue. **b** PA28α expression pattern in 158 oral carcinoma clinical samples. IHC staining showed the expression of PA28α increased paralleled with progression from normal epithelium to premalignant dysplasia to invasive carcinoma by using polyclonal antibody to PA28α. **c** PA28α expression correlates with OSCC prognosis in 98 clinical OSCC samples. IHC staining showed those cases with poorer prognosis exhibited stronger staining. *a*, a moderate-differentiated OSCC sample with disease free survival. *b*, a well-differentiated OSCC sample with regional neck recurrence within 2 years postoperation. *c*, a well-differentiated OSCC sample with cancer-related death with 4 years postoperation. The scale bar represent 100 μm

Our previously study with a small cohort indicated the potential importance of PA28α upregulation in OSCC [13, 14]. Our findings was supported by the Oncomine data analysis of two different reports [15, 16], which showed that the amount of PA28α mRNA in oral cancer tissues was significantly higher than normal tissues (Fig. 1a). Here, we employed a large clinical cohort of 158 tumor specimens, including 98 OSCC tumors, as well as 30 normal mucosa samples, and 30 precanceous oral leukoplakias to further define the role of PA28α in OSCC carcinogenesis. The common characteristics of these cohorts were summarized in Additional file 1: Table S1. The scoring scale was assessed referring to the previous studies [17, 18]. Briefly, staining intensities (scale, 1–3) and percentage of tumor staining (scale, 1–3) were determined independently, and total staining scale was expressed as a product of the two numbers (resulting in a staining scale of 1–9). For the determination of the potential prognostic value of PA28α expression in this cohort, patients with various tissue staining scale were grouped into 2 groups: group 1 (the staining scale ≤ 6) and group 2 (the staining scale equal to 6 to 9). Ectopic depletion systems were used to control antibody and staining-specificity Immunoreactivity of PA28α was readily detected in the cytoplasm and positive staining was rarely detected in normal epithelium (Additional file 2: Figure S2). We found that with increased severity of epithelial hyperplasia, the positive staining would spread into the epithelium with enhanced intensity (Fig. 1b).

### Expression levels of PA28α correlated with OSCC recurrence by using Univariate and Multivariate Cox Regression analysis

**Fig. 2:**
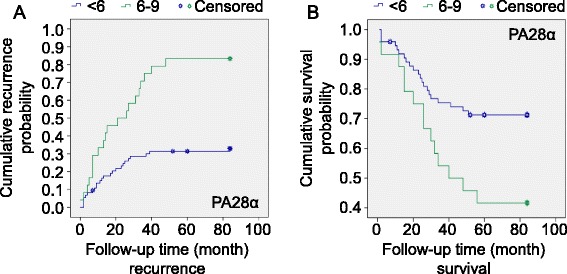
PA28α may be a discriminator of recurrence and survival in patients with OSCC defined by the Kaplan-Meier curves. **a** The curves showed that the recurrence in the subtypes divided by PA28α*;* **b** The curves showed that the survival in the subtypes divided by PA28α, *P < 0.05, P Values among different PA28α staining values were calculated by the log-rank test

Tissue slides were prepared from 98 OSCC cases. Those cases exhibiting strong staining (category 2) showed positive recurrence related indexes like higher cumulative recurrence probability (83.3 % vs. 32.9 %) (Additional file 3: Table S2 and Fig. 1c). Recurrence versus non-recurrence of the malignant lesions was considered to be one of the surrogates for clinical outcome of OSCC patients. Inclusion of all well-known factors with prognostic significance in our study would strengthen the utility of PA28α as a prognostic predictor alone or in combination with other factors. In Univariate Cox Regression analysis, PA28α staining (intensity, domain, category), together with smoking, clinical stage, differentiation, T-stage, and lymph node metastasis were all associated with an increased risk of recurrence (Table 1). Kaplan-Meier cumulative recurrence curves related to the above five factors demonstrated significant discrimination ability (Fig. 2 and Additional file 4: Figure S2).

**Table 1:** Univariate Cox Regression analysis on factors for OSCC recurrence

Variables	β	SE	Wald *χ* ^2^	*P* Value	*RR* (95 % *CI*)^a^
Alpha domain	0.543	0.240	5.111	0.024	1.722 (1.075-2.758)
Alpha intensity	0.868	0.245	12.864	0.000	2.382 (1.472-3.854)
Alpha (D×I)	0.256	0.064	15.889	0.000	1.292 (1.139-1.466)
Alpha (categorical)	1.298	0.307	17.923	0.000	3.664 (2.008-6.683)
Smoking	1.210	0.326	13.812	0.000	3.354 (1.772-6.349)
Current smoking	0.940	0.362	6.754	0.009	2.560 (1.260-5.200)
Smoking dose	0.001	0.000	4.943	0.026	1.001 (1.000-1.002)
Differentiation (3 vs. 1)	1.567	0.367	18.230	0.000	4.792 (2.334-9.837)
T-stage (3 vs. 1)	1.848	0.551	11.250	0.001	6.349 (2.156-18.698)
Lymphatic metastasis	0.704	0.302	5.420	0.020	2.021 (1.118-3.656)
Clinical stage (3 vs. 1)	1.737	0.746	5.422	0.020	5.679 (1.316-24.501)
Clinical stage (4 vs. 1)	2.230	0.762	8.570	0.003	9.300 (2.090-41.387)

^a^RR: relative risk, CI: confidence interval

Next, we used a multivariate regression analysis based on the Cox proportional hazard model to test the independent value of each parameter in predicting recurrence. The results showed that PA28α (P = 0.022, RR = 2.229, 95 % CI: 1.121-4.432), differentiation (P = 0.002, RR = 3.335, 95 % CI: 1.577-7.053) and lymphatic metastasis (P = 0.019, RR = 3.547, 95 % CI: 1.234-10.200) significantly influenced the recurrence rate independently (Table 2).

**Table 2:** Multivariate Cox Regression analysis on factors for OSCC recurrence

Variables	β	SE	Wald *χ* ^2^	*P* Value	*RR* (95 % *CI*)
Alpha (2 vs.1)	0.801	0.351	5.220	0.022	2.229 (1.121-4.432)
Differentiation (3 vs. 1)	1.204	0.382	9.932	0.002	3.335 (1.577-7.053)
Lymphatic metastasis	1.266	0.539	5.522	0.019	3.547 (1.234-10.200)

### Expression of PA28α correlated with OSCC survival by using Univariate and Multivariate Cox Regression analysis

The same cohort was used for survival analysis. Similar to recurrence those cases exhibiting strong staining scale (category 2) showed positive survival related indexes, such as lower cumulative survival probability (41.7 % vs. 71.3 %) (Additional file 5: Table S3 and Fig. 1c). In Univariate Cox Regression analysis, PA28α staining (intensity, domain, category), together with smoking, clinical stage, differentiation, T-stage, lymph node metastasis, and chemotherapy were all associated with an increased risk of death(P < 0.05, Table 3). Kaplan-Meier cumulative survival curves related to the above five factors (differentiation excluded) also demonstrated significant discrimination ability (Fig. 2 and Additional file 6: Figure S3).

**Table 3:** Univariate Cox Regression analysis on factors for OSCC survival

Variables	β	SE	Wald *χ* ^2^	*P* Value	*RR* (95 % *CI*)
Alpha domain	0.275	0.258	1.134	0.287	1.317 (0.794-2.186)
Alpha intensity	0.735	0.267	7.547	0.006	2.085 (1.234-3.521)
Alpha (D×I)	0.166	0.072	5.362	0.021	1.181 (1.026-1.359)
Alpha (categorical)	0.860	0.346	6.193	0.013	2.364 (1.201-4.655)
Smoking	1.310	0.366	12.806	0.000	3.704 (1.808-7.589)
Differentiation (2 vs. 1)	0.890	0.386	5.324	0.021	2.435 (1.143-5.185)
Differentiation (3 vs. 1)	0.937	0.450	4.332	0.037	2.552 (1.056-6.165)
T-stage (3 vs. 1)	1.453	0.641	5.142	0.023	4.278 (1.218-15.024)
Lymphatic metastasis	0.549	0.226	5.904	0.015	1.732 (1.112-2.697)
Clinical stage (4 vs.1)	1.714	0.782	4.803	0.028	5.553 (1.199-25.726)
Chemotherapy (no vs. yes)	0.839	0.357	5.532	0.019	2.313 (1.150-4.652)

Some factors (P < 0.20) in univariable logistic regression were included in the multivariable step-wise logistic regression model and fixed. PA28α (P = 0.058, RR = 2.190, 95 % CI: 0.975-4.918), differentiation (P = 0.014, RR = 2.819, 95 % CI: 1.233-6.444), T-stage (3 vs. 1) (P = 0.088, RR = 7.164, 95 % CI: 0.745-68.847), lymphatic metastasis (P = 0.004, RR = 5.397, 95 % CI: 1.694-17.194) and chemotherapy (P = 0.000, RR = 4.512, 95 % CI: 2.010-10.124) resulted as independent prognostic indicators associated with an increased risk of death (Table 4).

**Table 4:** Multivariate Cox Regression analysis on factors for OSCC recurrence

Variables	β	SE	Wald *χ* ^2^	*P* Value	*RR* (95 % *CI*)
Alpha (2 vs. 1)	0.784	0.413	3.603	0.058	2.190 (0.975-4.918)
Differentiation (2 vs. 1)	1.036	0.422	6.034	0.014	2.819 (1.233-6.444)
Differentiation (3 vs. 1)	0.826	0.475	3.025	0.082	2.285 (0.900-5.798)
T-stage (3 vs. 1)	1.969	1.115	2.909	0.088	7.164 (0.745-68.847)
Lymphatic metastasis	1.686	0.591	8.130	0.004	5.397 (1.694-17.194)
Chemotherapy (no vs. yes)	1.507	0.412	13.347	0.000	4.512 (2.010-10.124)

### PA28α knockdown inhibited cell growth without inducing apoptosis in OSCC cells *in vitro*

**Fig. 3:**
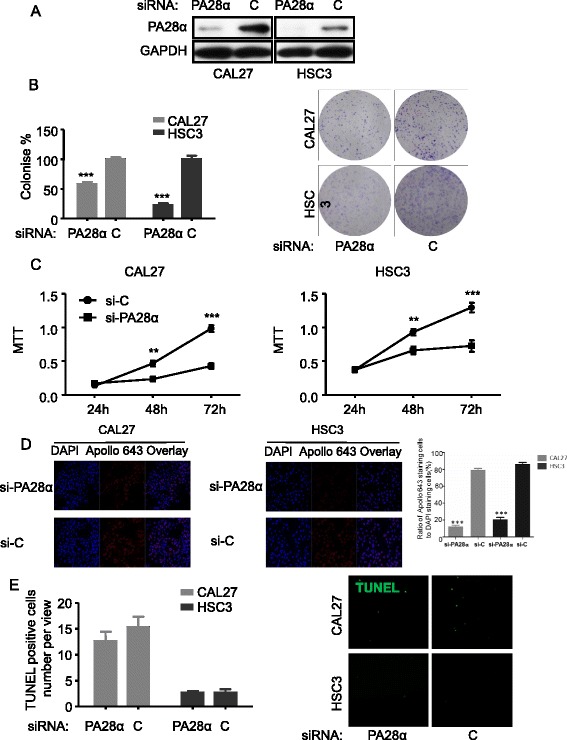
PA28α knockdown in CAL27 and HSC3 inhibits cell growth, but doesn’t induce apoptosis in vitro. **a** PA28α was knocked down by siRNA targeting PA28α, illustrated by Western blotting. **b** MTT assay, PA28α silencing in CAL27 and HSC3 inhibits cell growth. **c** Clonogenic assay, PA28α silencing in CAL27 and HSC3 inhibits clonogenic. **d** Edu assay, PA28α silencing in CAL27 and HSC3 inhibits proliferation. **e** TUNEL assay, PA28α silencing in CAL27 and HSC3 has no effect on apoptosis. PA28α overexpression in OSCC cell lines promotes cell proliferation in vitro was indicated in Additional file 8: Figure S5. Data are reported as means ± SEM for three independantly experiments. (ANOVA or T test, *p < 0.05, ** p < 0.01, *** p < 0.001)

Three PA28α targeting siRNAs were designed to downregulate PA28α expression. Two PA28α overexpressing OSCC cell lines, CAL27 and HSC3, were transfected with the most effective siRNA-PA28α while PA28 β and γ weren't affected (Additional file 7: Figure S4), following with tests of cells and subjected to MTT, colony formation, Edu coporation assay and TUNEL assay. PA28 knockdown (Fig. 3a) in CAL27 and HSC3 decreased cells viability, colony formation and cells proliferation (Fig. 3b, c, d), but no effects on apoptosis of these cells (Fig. 3e).

### PA28α knockdown inhibited migration, invasion in OSCC cells *in vitro*

**Fig. 4:**
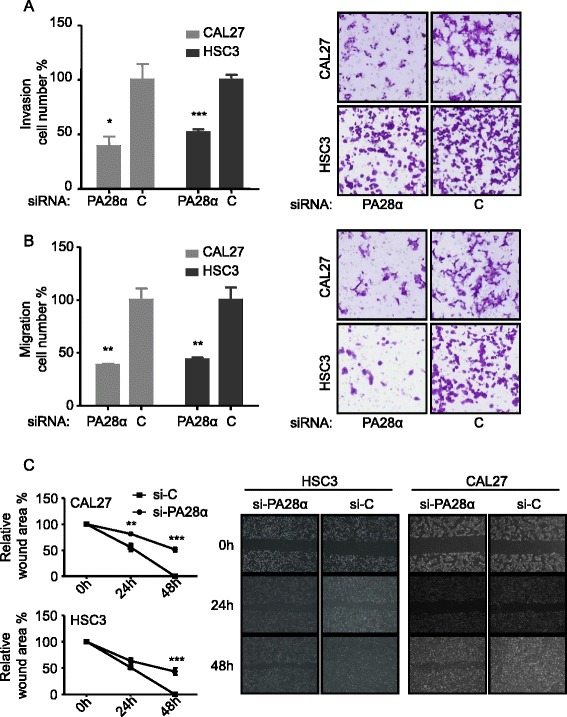
PA28α knockdown in CAL27 and HSC3 suppresses cell invasion, migration and wound healing properties in vitro. **a** Matrigel invasion assay, PA28α knockdown in CAL27 and HSC3 inhibits cell invasion. **b** Transwell migration assay, PA28α knockdown in CAL27 and HSC3 inhibits cell migration. **c** Wound healing assay, scratch wound of PA28α knockdown in CAL27 and HSC3 cells were not completely closed in 48 h, whereas cells transfected with siRNA-Control the scratch wound were successfully closed in 48 h. Data are reported as means ± SEM for three independantly experiments. (ANOVA or T test,, * p < 0.05, ** p < 0.01, *** p < 0.001)

Tumor cell metastasis is a multistep process, including migration and invasion to allow dissemination of tumor cells from the primary tumor site. To test whether PA28α knockdown altered the invasiveness and migration of OSCC cells, transwell migration and invasion assay, wound healing assay were employed. OSCC cells with PA28α knockdown showed a consistent and statistically significant reduction of invasion ability and migration ability. The invasion of CAL27 and HSC3 with PA28α knockdown was reduced to 40 % and 52 % respectively in transwell invasion assay (Fig. 4a). The migration of CAL27 and HSC3 with PA28α knockdown was reduced to 39 % and 44 %, respectively in transwell migration assay (Fig. 4b). The scratch wound of PA28α knockdown groups were not completely closed by 48 hours compared to the control cells (Fig. 4c).

### PA28α silencing suppressed *in vivo* tumor growth of OSCC cells

**Fig. 5:**
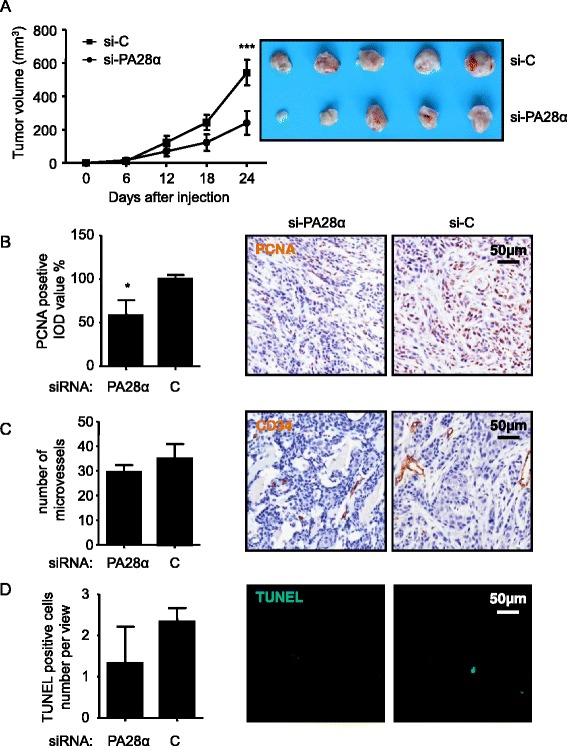
PA28α knockdown significantly inhibits tumor growth through its effects on cell proliferation but not on angiogenesis and apoptosis *in vivo*. **a** The tumor volumes of HSC3 cells with the transfection of chemically modified (both 2’ OMe and 3’ Chol) siRNA-PA28α, were remarkably decreased, comparing to the siRNA-Control groups in BALB-C nude mice after 24 days (mean ± SEM, n = 6). **b** IOD of PCNA-positive tumor cells of siRNA-PA28α group was significantly lower than the siRNA-Control group. **c** No significant difference of CD34 staining between in siRNA-PA28α group and in siRNA-Control group. **d** No significant difference of TUNEL staining between in siRNA-PA28α group and in siRNA-Control group. Data are reported as means ± SEM for three independantly experiments. (ANOVA or T test,, * p < 0.05, ** p < 0.01, *** p < 0.001)

Next, we examined the role of PA28α in tumor formation and tumor growth *in vivo*. HSC3 cells were treated with 2’ OMe and 3’ Chol modified siRNA-PA28α (the same siRNA sequence as previous *in vitro* experiments) to knockdown PA28α. The ability of these PA28 α knockdown OSCC cells to form tumors as xenografts in BALB/C nude mice was examined. It is known that chemical modification of the 2’-O-methyl (2’OMe) can indirectly improve nuclease resistance of the internucleotide phosphate bond in siRNA and at the same time can increase duplex stability, and may also provide protection of siRNA from immune activation. Cholesterol conjugation (3’ Chol) of siRNAs has been demonstrated to have an important role for systemic activity *in vivo*, which could provide further opportunity to optimize systemic activity through chemical conjugation [19]. Successful knockdown of PA28α with this new synthetic/modified siRNA-PA28α was not shown. As shown in Fig. 5a, there was a 56 % reduction of tumor volume in the siRNA-PA28α tumors comparing to that of the siRNA-Control tumors. The reduction in tumor growth in the siRNA-PA28α tumors was accompanied by a significant reduction of PCNA expression (58 % reduction by PCNA staining) comparing to that of the siRNA-Control tumors (Fig. 5b). There was no difference in angiogenesis and apoptosis between the two groups (by CD34 staining and TUNEL staining respectively, Fig. 5c and d). Thus, PA28α is involved OSCC tumor growth *in vivo*.

## Discussion

In our preliminary proteomics analysis, PA28α was found to be differentially expressed between OSCC tissues and normal epithelium [20]. Here we examined the role of PA28α in clinical prognosis and OSCC tumorigenesis. The association between PA28α expression and survival and recurrence of OSCC was evaluated, and most of the common factors such as epidemiological and clinic-pathological factors were included in the regression model. It has been shown that PA28α expression may be an independent predictor for poorer patient outcomes with increased risk of relapse and death (p < 0.05). The immunoproteasome is a large proteolytic machinery derived from the constitutive proteasome (also known as the 26S proteasome) and is expressed abundantly in antigen-presenting immune cells [21, 22]. The immunoproteasome plays a critical role in immune system because it degrade intracellular proteins which are further presented by major histocompatibility complex (MHC) class I molecules. As the main component of immunoproteosome, PA28α form either homoheptamers or heteroheptamers with PA28β in cells to participate in MHC class I antigen presentation to CD8+ T lymphocytes [23, 24]. Interestingly, various roles for the immunoproteasome in nonimmune cells have been reported recently [25–27], suggesting that there could be unknown roles for the immunoproteasome. Recently, it has been shown that PA28α might be overexpressed in multiple cancers, including ovarian cancer and prostate cancer [11, 12], but its role in cancer biology remained poorly defined. Our results suggested PA28α is associated with regulation of proliferation, migration and invasion of OSCC cells in vitro and tumor generation in vivo (BALB-C nude mice, the animal lacks a thymus, is unable to produce T-cells, and is therefore immunodeficient). Interestingly, we can't bypass the possible effect from immune system in immunodeficient mice model, but only assesses the contribution of PA28α to OSCC tumor generation. Our current study shows that PA28α may be an important inhibition target for tumor generation. On the other hand, PA28αβ-deficient mice has been showed bred well, apparently healthy and normal immune responses against influenza A virus infection [28], which suggests that PA28αβ inhibition may not significantly affect the normal health and growth of mice. Moreover, PA28α was also considered to have potential role in inducing anti-tumor immunity, as demonstrated in colon cancer cells and cervical carcinoma model [29, 30]. Thus, it is possible that PA28α is a potential effective, safe therapeutic target for OSCC.

It has been showed that inflammatory cytokines could induce the expression of five subunits of constitutive proteasome (iβ1 [LMP2], iβ2 [LMP10], iβ5 [LMP7], PA28α, and PA28β), which assemble on the proteasome core to create the immunoproteasome [31, 32], and this immunoproteasome might have an important role in mediating OSCC tumor generation. Such scenario could be at least partially supported by our current finding suggesting that that PA28α was an key oncogenic factor of OSCC. These studies make a possible link between inflammatory cytokines and OSCC happen or development. On the other hand, it’s still unclear that PA28α is a driver factor or a passenger enhancer during OSCC development and progress.

The potential mechanistic involvement of PA28α in tumorigenesis and metastasis remain unclear. However, a recent study in Burkitt's lymphoma cells showed that induction of the EBV lytic cycle increased the expression of the proteasomal components, including β2, β1i, PA28α and PA28β [33]. Although little is known about the factors regulating the proteasome structures in cells and their activity, the composition and function of the 26S proteasome including PA28α can be changed by expression of the EGFRvIII [34]. Moreover, some studies reported that Nitric Oxide could inhibit neointimal hyperplasia via reduction of PA28α in the vasculature, which therefore could facilitate the protection from detrimental effect ofoxidative stress. Also, future in-depth study to determine the involvement and mechanisms of PA28α-linked oxidative stress in cancer development and progress are required which suggest oxidative stress might be an important area for the future PA28α research in cancer [35, 36].

## Conclusion

In conclusion, our study demonstrated that PA28α has a critical role in tumorigenesis and development. Silencing of PA28α suppresses OSCC cell growth and metastasis in vitro and suppresses OSCC growth in vivo. Together, PA28α can be considered as a potential prognosis predictor, as well as a potential therapeutic drug target.

### Ethics, consent and permissions

All of the patients or their relatives gave informed consent for the use of their tissue in the experimental procedures prior to their inclusion in the study. The project was approved by the Scientific and Ethical Committee of Sichuan University, China.

## Additional files

Additional file 1: Table S1. Common characteristics of study population (n=98). (DOCX 16 kb) Additional file 2: Figure S2. Immunocellularchemistry analysis of PA28α on CAL27 (A) and HSC3 (B) cell lines treated with si-PA28a or si-Control. PA28α was significantly knocked down with siRNA and localized predominantly on the cytoplasm, sporadically on the nucleus. (PPT 1 mb) Additional file 3: Table S2. Recurrence related indexes based on different subgroups. (DOCX 18 kb) Additional file 4: Figure S2. The curves showed that the recurrence in the subtypes divided by T-stage *(A)* , Smoking *(B)*,Lymphatic metastasis *(C)* and Differentiation *(D)* were well separated. (PPT 244 kb) Additional file 5: Table S3. Survival related indexes based on different subgroups. (DOCX 18 kb) Additional file 6: Figure S3. The curves showed that the survival in the subtypes divided by T-stage *(A),* Smoking *(B)* and Chemotherapy *(C)* and Lymphatic metastasis *(D)* were well separated. **P < 0.05, P* Values were calculated by the log-rank test. (PPT 232 kb) Additional file 7: Figure S4. PA28α, β,γexpression after PA28α knock down. The results of qPCR showed PA28α expression was significantly decreased in CAL27 and HSC6 cells after transfection with PA28α specific siRNA. PA28 β and γ weren't affected in these cells. (PPT 157 kb) Additional file 8: Figure S5. PA28α overexpression in OSCC cell lines promotes cell proliferation in vitro. PA28α overexpression stable OSCC cell lines (CAL27 and HSC3 cell) were assessed for cell proliferation by EdU assay. CAL27 and HSC3 cell morphology was assessed by laser scanning confocal microscopy (200×). After 24 hours transfection, cells were stained with Edu (Apoll 643, red color) and DAPI. Data are reported as means ± SEM for three independent experiments. A, PA28α overexpression in CAL27 and HSC3 cells was illustrated by Western blotting. B&C, Edu assay. (PPT 241 kb)

## Abbreviations

OSCC: oral squamous cell carcinoma
CP: core particle
EdU: 5-ethynyl-2'-deoxyuridine

## References

[1] Neville BW, Day TA (2002). Oral cancer and precancerous lesions. CA Cancer J Clin.

[2] Lewin F, Norell SE, Johansson H, Gustavsson P, Wennerberg J, Biorklund A, Rutqvist LE. Smoking tobacco, oral snuff, and alcohol in the etiology of squamous cell carcinoma of the head and neck: a population-based case-referent study in Sweden. Cancer. 1998;82:1367–75. 10.1002/(sici)1097-0142(19980401)82:7<1367::aid-cncr21>3.0.co;2-3 9529030

[3] Scully C (2002). Oral squamous cell carcinoma; from an hypothesis about a virus, to concern about possible sexual transmission. Oral oncology.

[4] Rechsteiner M, Hill CP (2005). Mobilizing the proteolytic machine: cell biological roles of proteasome activators and inhibitors. Trends in cell biology.

[5] Masters EI, Pratt G, Forster A, Hill CP (2005). Purification and analysis of recombinant 11S activators of the 20S proteasome: Trypanosoma brucei PA26 and human PA28 alpha, PA28 beta, and PA28 gamma. Methods in enzymology.

[6] Li X, Lonard DM, Jung SY, Malovannaya A, Feng Q, Qin J, Tsai SY, Tsai MJ, O'Malley BW. The SRC-3/AIB1 coactivator is degraded in a ubiquitin- and ATPindependent manner by the REGgamma proteasome. Cell. 2006;124:381–92. 10.1016/j.cell.2005.11.037 16439211

[7] Li X, Amazit L, Long W, Lonard DM, Monaco JJ, O'Malley BW (2007). Ubiquitin- and ATP-independent proteolytic turnover of p21 by the REGgamma-proteasome pathway. Mol. Cell.

[8] Li L, Dang Y, Zhang J, Yan W, Zhai W, Chen H, Li K, Tong L, Gao X, Amjad A, Ji L, Jing T, Jiang Z, Shi K, Yao L, Song D, Liu T, Yang X,Yang C, Cai X, Xu W, Huang Q, He J, Liu J, Chen T, Moses RE, Fu J, Xiao J, Li X. REGγ is critical for skin carcinogenesis by modulating the Wnt/β-catenin pathway. Nat Commun. 2015;6:6875. 10.1038/ncomms7875 25908095

[9] Groettrup M, Soza A, Eggers M, Kuehn L, Dick TP, Schild H, Rammensee HG, Koszinowski UH, Kloetzel PM. A role for the proteasome regulator PA28alpha in antigen presentation. Nature. 1996;381:166–8. 10.1038/381166a0 8610016

[10] Murata S, Udono H, Tanahashi N, Hamada N, Watanabe K, Adachi K, Yamano T, Yui K, Kobayashi N, Kasahara M, Tanaka K, Chiba T. Immunoproteasome assembly and antigen presentation in mice lacking both PA28alpha and PA28beta. EMBO J. 2001;20:5898–907. 10.1093/emboj/20.21.5898 PMC125708 11689430

[11] Longuespee R, Boyon C, Castellier C, Jacquet A, Desmons A, Kerdraon O, Vinatier D, Fournier I, Day R, Salzet M. The C-terminal fragment of the immunoproteasome PA28S (Reg alpha) as an early diagnosis and tumor-relapse biomarker: evidence from mass spectrometry profiling. Histochem Cell Biol. 2012;138:141–54. 10.1007/s00418-012-0953-0 22532226

[12] Lemaire R, Menguellet SA, Stauber J, Marchaudon V, Lucot JP, Collinet P, Farine MO, Vinatier D, Day R, Ducoroy P, Salzet M, Fournier I. Specific MALDI imaging and profiling for biomarker hunting and validation: fragment of the 11S proteasome activator complex, Reg alpha fragment, is a new potential ovary cancer biomarker. J Proteome Res. 2007;6:4127–34. 10.1021/pr0702722 17939699

[13] Wang Z, Feng X, Liu X, Jiang L, Zeng X, Ji N, Li J, Li L, Chen Q. Involvement of potential pathways in malignant transformation from oral leukoplakia to oral squamous cell carcinoma revealed by proteomic analysis. BMC genomics. 2009;10:383. 10.1186/1471-2164-10-383 PMC2746235 19691830

[14] Wang Z, Jiang L, Huang C, Li Z, Chen L, Gou L, Chen P, Tong A, Tang M, Gao F. Comparative proteomics approach to screening of potential diagnostic and therapeutic targets for oral squamous cell carcinoma, vol. 7: ASBMB, 2008:1639. 10.1074/mcp.M700520-MCP200 PMC2556024 18458027

[15] Talbot SG, Estilo C, Maghami E, Sarkaria IS, Pham DK, Oc P, Socci ND, Ngai I, Carlson D, Ghossein R, Viale A, Park BJ, et al. Gene expression profiling allows distinction between primary and metastatic squamous cell carcinomas in the lung. Cancer research. 2005;65:3063–71. 10.1158/0008-5472.CAN-04-1985 15833835

[16] Pyeon D, Newton MA, Lambert PF, den Boon JA, Sengupta S, Marsit CJ, Woodworth CD, Connor JP, Haugen TH, Smith EM, Kelsey KT, Turek LP (2007). Fundamental differences in cell cycle deregulation in human papillomavirus-positive and human papillomavirus-negative head/neck and cervical cancers. Cancer research.

[17] Tan F, Zhu H, Tao Y, Yu N, Pei Q, Liu H, Zhou Y, Xu H, Song X, Li Y, Zhou Z, He X, Zhang X, Pei H. Neuron navigator 2 overexpression indicates poor prognosis of colorectal cancer and promotes invasion through the SSH1L/cofilin-1 pathway. J Exp Clin Cancer Res. 2015;34(1):117. 10.1186/s13046-015-0237-3 PMC4600204 26452645

[18] Kreisberg JI, Malik SN, Prihoda TJ, Bedolla RG, Troyer DA, Kreisberg S, Ghosh PM. Phosphorylation of Akt (Ser473) is an excellent predictor of poor clinical outcome in prostate cancer. Cancer Res. 2004;64(15):5232–6. 10.1158/0008-5472.CAN-04-0272 15289328

[19] Soutschek J, Akinc A, Bramlage B, Charisse K, Constien R, Donoghue M, Elbashir S, Geick A, Hadwiger P, Harborth J. Therapeutic silencing of an endogenous gene by systemic administration of modified siRNAs. Nature. 2004;432:173–8. 10.1038/nature03121 15538359

[20] Wang Z, Jiang L, Huang C, Li Z, Chen L, Gou L, Chen P, Tong A, Tang M, Gao F, Shen J, Zhang Y, et al. Comparative proteomics approach to screening of potential diagnostic and therapeutic targets for oral squamous cell carcinoma. Mol cell proteomic. 2008;7:1639–50. 10.1074/mcp.M700520-MCP200 PMC2556024 18458027

[21] Haorah J, Heilman D, Diekmann C, Osna N, Donohue Jr TM, Ghorpade A, Persidsky Y. Alcohol and HIV decrease proteasome and immunoproteasome function in macrophages: implications for impaired immune function during disease. Cell Immunol. 2004;229:139–48. 10.1016/j.cellimm.2004.07.005 15474528

[22] Frisan T, Levitsky V, Masucci MG (2000). Variations in proteasome subunit composition and enzymatic activity in Blymphoma lines and normal B cells,”. Int J Cancer..

[23] Murata S, Udono H, Tanahashi N, Hamada N, Watanabe K, Adachi K, Yamano T, Yui K, Kobayashi N, Kasahara M, Tanaka K, Chiba T. Immunoproteasome assembly and antigen presentation in mice lacking both PA28α and PA28β. EMBO J. 2001;20:5898-907. 10.1093/emboj/20.21.5898 PMC125708 11689430

[24] Groettrup M, van den Broek M, Schwarz K, Macagno A, Khan S, de Giuli R, Schmidtke G. Structural plasticity of the proteasome and its function in antigen processing. Crit Rev Immunol. 2001;21:339–58. 11922078

[25] Shachar I, Karin N (2011). The dual roles of inflammatory cytokines and chemokines in the regulation of autoimmune diseases and theirclinical implications. J Leukoc Biol.

[26] Ahn JY, Tanahashi N, Akiyama K, Hisamatsu H, Noda C, Tanaka K, Chung CH, Shibmara N, Willy PJ, Mott JD PJ, et al. Primary structures of two homologous subunits of PA28, a gamma interferon-inducible protein activator of the 20S proteasome. FEBS Lett. 1995;366:37–42. 10.1016/0014-5793(95)00492-r 7789512

[27] Tanahashi N, Yokota K, Ahn JY, Chung CH, Fujiwara T, Takahashi E, DeMartino GN, Slaughter CA, Toyonaga T, Yamamura K, Shimbara N, Tanaka K. Molecular properties of the proteasome activator PA28 family proteins and $$ \gamma $$-interferon regulation,”. Genes Cells. 1997;2:195–211. 10.1046/j.1365-2443.1997.d01-308.x 9189757

[28] Zhang M, Ishii K, Hisaeda H, Murata S, Chiba T, Tanaka K, Li Y, Obata C, Furue M, Himeno K. Ubiquitin-fusion degradation pathway plays an indispensable role in naked DNA vaccination with a chimeric gene encoding a syngeneic cytotoxic T lymphocyte epitope of melanocyte and green fluorescent protein. Immunology. 2004;112:567–74. 10.1111/j.1365-2567.2004.01916.x PMC1782537 15270727

[29] Miyagi T, Tatsumi T, Takehara T, Kanto T, Kuzushita N, Sugimoto Y, Jinushi M, Kasahara A, Sasaki Y, Hori M, Hayashi N. Impaired expression of proteasome subunits and human leukocyte antigens class I in human colon cancer cells. J Gastroenterol Hepatol. 2003;18:32–40. 10.1046/j.1440-1746.2003.02921.x 12519221

[30] Ritz U, Momburg F, Pilch H, Huber C, Maeurer MJ, Seliger B (2001). Deficient expression of components of the MHC class I antigen processing machinery in human cervical carcinoma. Int J Oncol.

[31] Kloetzel PM, Ossendorp F (2004). Proteasome is required for class I-restricted presentation by Fcgamma receptor-mediated endocytosis in primary biliary cirrhosis. Curr Opin Immunol..

[32] Tanaka K (2009). The proteasome: overview of structure and functions. Proc Jpn Acad Ser B Phys Biol Sci..

[33] De Leo A, Matusali G, Arena G, Di Renzo L, Mattia E (2010). Epstein-Barr virus lytic cycle activation alters proteasome subunit expression in Burkitt's lymphoma cells. Biol Chem..

[34] Kim K, Brush JM, Watson PA, Cacalano NA, Iwamoto KS, McBride WH (2008). Epidermal growth factor receptor vIII expression in U87 glioblastoma cells alters their proteasome composition, function, and response to irradiation. Mol Cancer Res..

[35] Tsihlis ND, Kapadia MR, Vavra AK, Jiang Q, Fu B, Martinez J, Kibbe MR. Nitric oxide decreases activity and levels of the 11S proteasome activator PA28 in the vasculature. Nitric Oxide. 2012;27:50–8. 10.1016/j.niox.2012.04.006 22561112

[36] Yagi H, Tan W, Dillenburg-Pilla P, Armando S, Amornphimoltham P, Simaan M, Weigert R, Molinolo AA, Bouvier M, Gutkind JS. A synthetic biology approach reveals a CXCR4-G13-Rho signaling axis driving transendothelial migration of metastatic breast cancer cells. Science signaling. 2011;4:ra60. 10.1126/scisignal.2002221 PMC3429372 21934106

