# Supplementary material for: Overexpression of proteasomal activator PA28α serves as a prognostic factor in oral squamous cell carcinoma
Source: J Exp Clin Cancer Res. 2016 Feb 19;35:35. doi: 10.1186/s13046-016-0309-z (PMC4759779; doi:10.1186/s13046-016-0309-z)
Supplement: Additional file 1: Table S1. — Common characteristics of study population (n=98). (DOCX 16 kb) [file 13046_2016_309_MOESM1_ESM.docx]

**Supplementary Table 1. Common characteristics of study population (n=98)**

| variables | Statistic |
| --- | --- |
| Age (year) | 59.7±12.3 |
| Gender (Female %) | 33.7 |
| Smoking (%) | 44.9 |
| Current smoking (%) | 13.3 |
| Smoking dose (packet/year, *M* (*Q*_1_, *Q*_3_)) | 360 (180, 365) |
| Drinking (%) | 49.0 |
| Drinking dose (ml/year, *M* (*Q*_1_, *Q*_3_)) | 17250 (600, 36000) |
| Differentiation (%) |  |
| 1 | 56.1 |
| 2 | 28.6 |
| 3 | 15.3 |
| T-stage (%) |  |
| 1 | 20.4 |
| 2 | 54.1 |
| 3 | 20.2 |
| 4 | 5.3 |
| Lymphatic metastasis (%) |  |
| 0 | 65.4 |
| 1 | 25.5 |
| 2 | 10.2 |
| Clinical stage |  |
| 1 | 16.3 |
| 2 | 33.7 |
| 3 | 32.7 |
| 4 | 17.3 |
| Surgery |  |
| 1 | 42.9 |
| 2 | 52.0 |
| 3 | 5.1 |
| Radiotherapy (yes %) | 7.1 |
| Chemotherapy (yes %) | 51.0 |

*One subject without employment information
