# Supplementary figures and images for: Overexpression of proteasomal activator PA28α serves as a prognostic factor in oral squamous cell carcinoma
Source: J Exp Clin Cancer Res. 2016 Feb 19;35:35. doi: 10.1186/s13046-016-0309-z (PMC4759779; doi:10.1186/s13046-016-0309-z)

## Slide 1
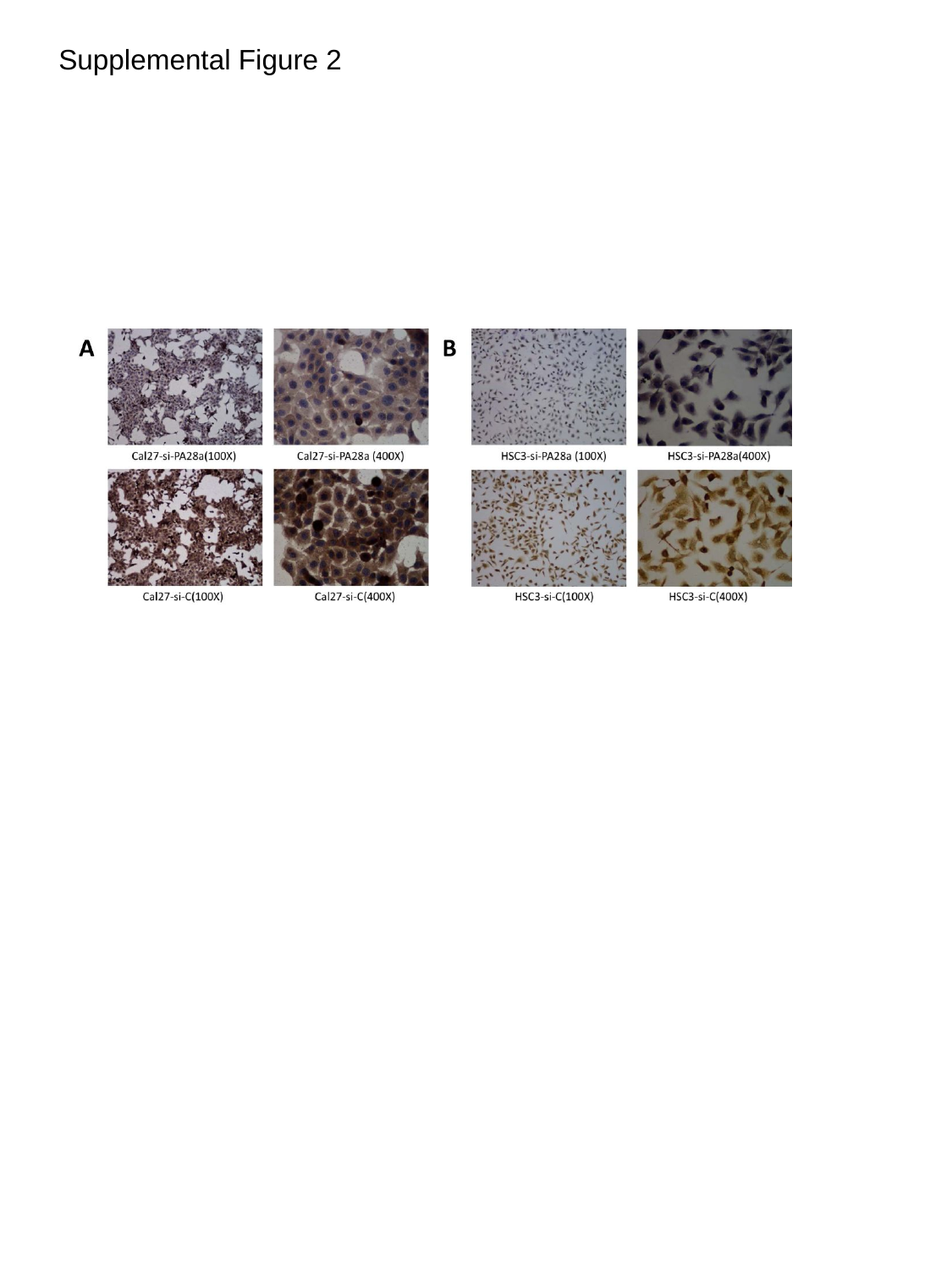

Supplemental Figure 2

Supplement: Additional file 2: Figure S2. — Immunocellularchemistry analysis of PA28α on CAL27 (A) and HSC3 (B) cell lines treated with si-PA28a or si-Control. PA28α was significantly knocked down with siRNA and localized predominantly on the cytoplasm, sporadically on the nucleus. (PPT 1 mb) [file 13046_2016_309_MOESM2_ESM.ppt]

## Slide 1
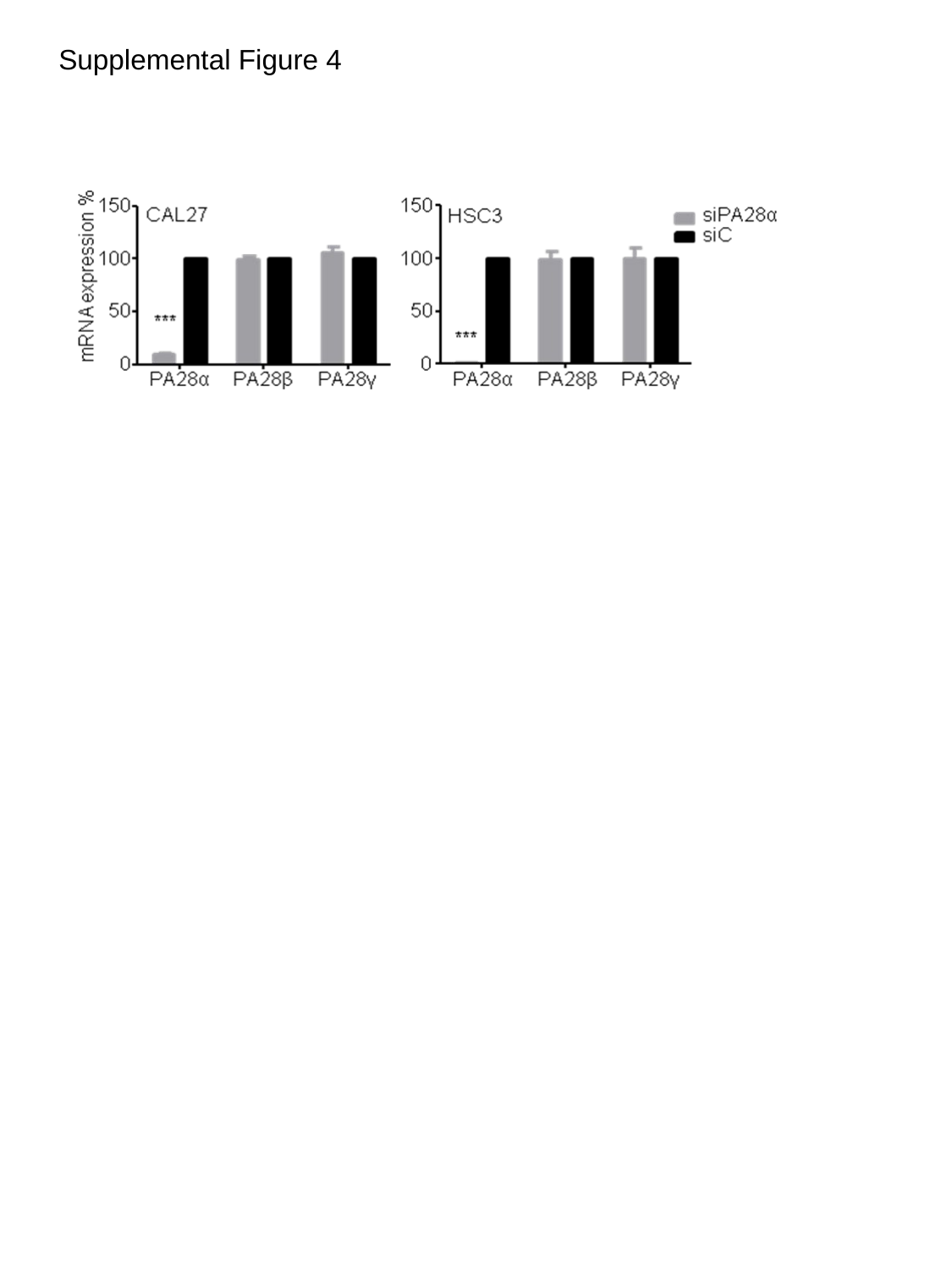

Supplemental Figure 4

Supplement: Additional file 7: Figure S4. — PA28α, β,γexpression after PA28α knock down. The results of qPCR showed PA28α expression was significantly decreased in CAL27 and HSC6 cells after transfection with PA28α specific siRNA. PA28 β and γ weren't affected in these cells. (PPT 157 kb) [file 13046_2016_309_MOESM7_ESM.ppt]

## Slide 1
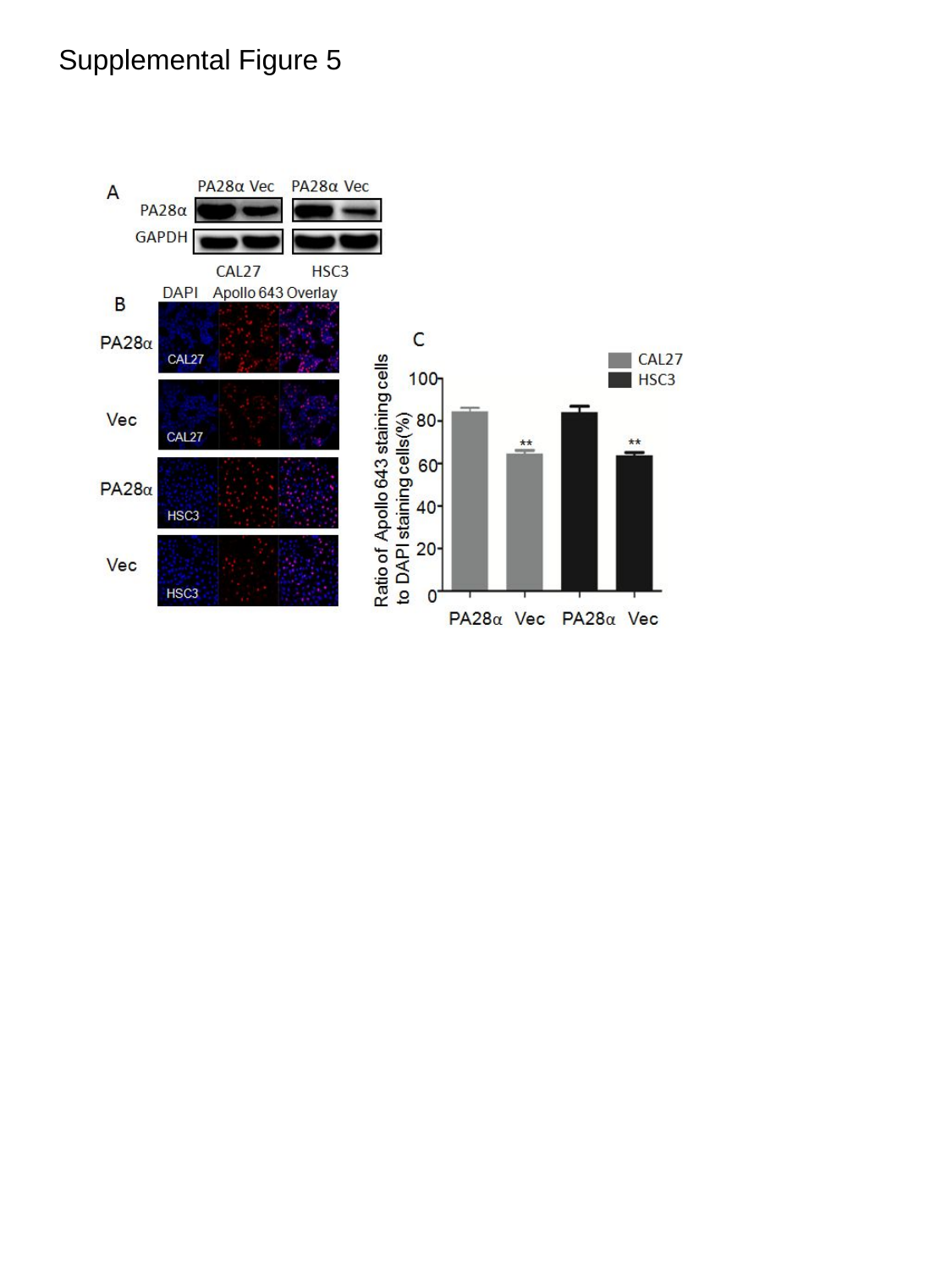

Supplemental Figure 5

Supplement: Additional file 8: Figure S5. — PA28α overexpression in OSCC cell lines promotes cell proliferation in vitro. PA28α overexpression stable OSCC cell lines (CAL27 and HSC3 cell) were assessed for cell proliferation by EdU assay. CAL27 and HSC3 cell morphology was assessed by laser scanning confocal microscopy (200×). After 24 hours transfection, cells were stained with Edu (Apoll 643, red color) and DAPI. Data are reported as means ± SEM for three independent experiments. A, PA28α overexpression in CAL27 and HSC3 cells was illustrated by Western blotting. B&C, Edu assay. (PPT 241 kb) [file 13046_2016_309_MOESM8_ESM.ppt]
