# Supplementary material for: Overexpression of proteasomal activator PA28α serves as a prognostic factor in oral squamous cell carcinoma
Source: J Exp Clin Cancer Res. 2016 Feb 19;35:35. doi: 10.1186/s13046-016-0309-z (PMC4759779; doi:10.1186/s13046-016-0309-z)
Supplement: Additional file 3: Table S2. — Recurrence related indexes based on different subgroups. (DOCX 18 kb) [file 13046_2016_309_MOESM3_ESM.docx]

Supplementary Table 2 Recurrence related indexes based on different subgroups

| Index^a^ | Total | Alpha | |  | Smoking | |  | Differentiation | | |  | T-stage | | |  | Lymphatic metastasis | |
| --- | --- | --- | --- | --- | --- | --- | --- | --- | --- | --- | --- | --- | --- | --- | --- | --- | --- |
|  |  | Group 1 | Group 2 |  | No | Yes |  | Group 1 | Group 2 | Group 3 |  | Group 1 | Group 2 | Group 3 |  | No | Yes |
|  | (n=98) | (n=74) | (n=24) |  | (n=54) | (n=44) |  | (n=55) | (n=28) | (n=15) |  | (n=20) | (n=53) | (n=25) |  | (n=63) | (n=35) |
| CRP (No.) | 45.3% (44) | 32.9% (24) | 83.3% (20) |  | 26.3% (14) | 68.2% (30) |  | 31.4% (17) | 46.4% (13) | 93.3% (14) |  | 20.6% (4) | 37.9% (20) | 80.0% (20) |  | 37.1% (23) | 60.0% (21) |
| WR (No.) | 4.1% (4) | 5.4% (4) | 0.0% (0) |  | 7.4% (4) | 0.0% (0) |  | 7.3% (4) | 0.0% (0) | 0.0% (0) |  | 10.0% (2) | 4.0% (2) | 0.0% (0) |  | 6.3% (4) | 0.0% (0) |
| MRT (*Q*_1_, *Q*_3_)^b^ | 14 (7, 27) | 13 (6, 25) | 15 (7, 32) |  | 10 (4, 20) | 18 (7, 28) |  | 15 (9, 24) | 9 (2, 31) | 18 (7, 27) |  | 11 (7, 20) | 15 (7, 36) | 13 (5, 27) |  | 18 (7, 27) | 13 (2, 25) |
| EMRT (SE)^b^ | 54 (4) | 62 (4) | 30 (6) |  | 66 (4) | 40 (5) |  | 63 (4) | 52 (7) | 26 (7) |  | 69 (7) | 61 (5) | 29 (6) |  | 61 (4) | 43 (6) |
| MFT (*Q*_1_, *Q*_3_)^b^ | 84 (15, 84) | 84 (24, 84) | 24 (7, 38) |  | 84 (39, 84) | 28 (12, 84) |  | 84 (24, 84) | 84 (11, 84) | 18 (7, 34) |  | 84 (43, 84) | 84 (27, 84) | 21 (9, 33) |  | 84 (24, 84) | 34 (9, 84) |
| TFT^b^ | 0~84 | 0~84 | 0~84 |  | 0~84 | 0~84 |  | 0~84 | 0~84 | 0~84 |  | 0~84 | 0~84 | 0~84 |  | 0~84 | 0~84 |
| aAbbreviations: CRP, Cumulative recurrence probability; WR, Withdrawal rate; MRT, Median recurrence time; EMRT, Estimated means for recurrence time; SE, std error; MFT, Median follow-up time; TFT, Total follow-up time | | | | | | | | | | | | | | | | | |
| ^b^month stated otherwise | | | | | | | | | | | | | | | | | |
