# Supplementary material for: Overexpression of proteasomal activator PA28α serves as a prognostic factor in oral squamous cell carcinoma
Source: J Exp Clin Cancer Res. 2016 Feb 19;35:35. doi: 10.1186/s13046-016-0309-z (PMC4759779; doi:10.1186/s13046-016-0309-z)
Supplement: Additional file 4: Figure S2. — The curves showed that the recurrence in the subtypes divided by T-stage (A) , Smoking (B),Lymphatic metastasis (C) and Differentiation (D) were well separated. (PPT 244 kb) [file 13046_2016_309_MOESM4_ESM.ppt]

## Slide 1
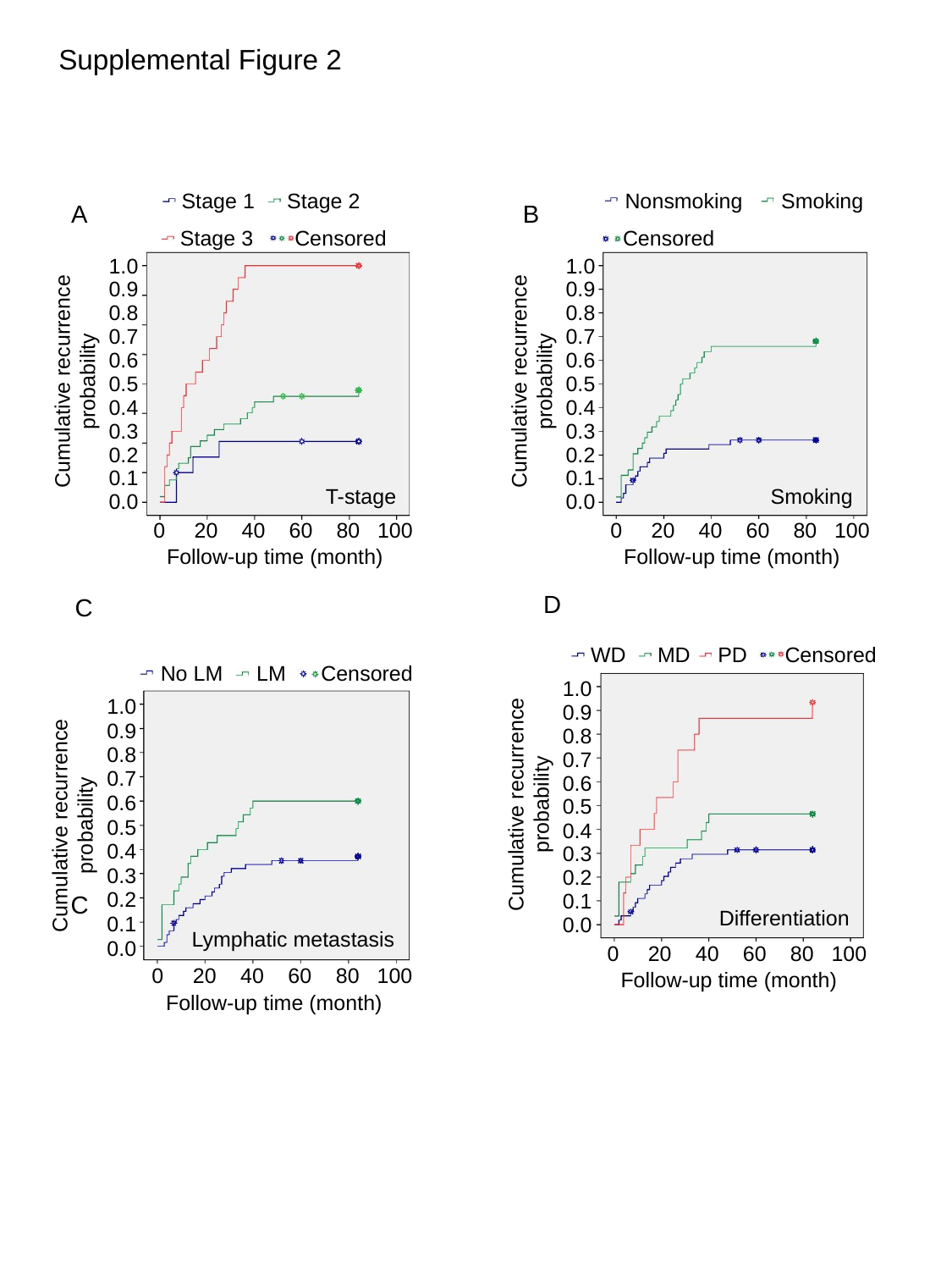

Supplemental Figure 2
Stage 1
Stage 2
Nonsmoking
Smoking
Stage 3
Censored
Censored
1.0
0.9
0.8
0.7
0.6
0.5
0.4
0.3
0.2
0.1
0.0
1.0
0.9
0.8
0.7
0.6
0.5
0.4
0.3
0.2
0.1
0.0
T-stage
Smoking
Cumulative recurrence probability
Cumulative recurrence probability
0
20
40
60
80
100
0
20
40
60
80
100
Follow-up time (month)
Follow-up time (month)
A
B
D
C
WD
MD
PD
Censored
1.0
0.9
0.8
0.7
0.6
0.5
0.4
0.3
0.2
0.1
0.0
Differentiation
Cumulative recurrence probability
0
20
40
60
80
100
Follow-up time (month)
No LM
LM
Censored
1.0
0.9
0.8
0.7
0.6
0.5
0.4
0.3
0.2
0.1
0.0
Cumulative recurrence probability
Lymphatic metastasis
0
20
40
60
80
100
Follow-up time (month)
C
