# Supplementary material for: Overexpression of proteasomal activator PA28α serves as a prognostic factor in oral squamous cell carcinoma
Source: J Exp Clin Cancer Res. 2016 Feb 19;35:35. doi: 10.1186/s13046-016-0309-z (PMC4759779; doi:10.1186/s13046-016-0309-z)
Supplement: Additional file 5: Table S3. — Survival related indexes based on different subgroups. (DOCX 18 kb) [file 13046_2016_309_MOESM5_ESM.docx]

Supplementary Table 3 Survival related indexes based on different subgroups

| Index^a^ | Total | Alpha | | Smoking | | Differentiation | | | T-stage | | | Lymphatic metastasis | | chemotherapy | |
| --- | --- | --- | --- | --- | --- | --- | --- | --- | --- | --- | --- | --- | --- | --- | --- |
|  |  | Group 1 | Group 2 | No | Yes | Group 1 | Group 2 | Group 3 | Group 1 | Group 2 | Group 3 | No | Yes | No | Yes |
|  | (n=98) | (n=74) | (n=24) | (n=54) | (n=44) | (n=55) | (n=28) | (n=15) | (n=20) | (n=53) | (n=25) | (n=63) | (n=35) | (n=48) | (n=50) |
| CSP (No.) | 63.9% (35) | 71.3% (21) | 41.7% (14) | 79.2% (11) | 45.5% (24) | 75.9% (13) | 50.0% (14) | 46.7% (8) | 84.2% (3) | 64.2% (19) | 48.0% (13) | 71.0% (18) | 51.4% (17) | 52.1% (23) | 75.5% (12) |
| WR (No.) | 4.1% (4) | 5.4% (4) | 0.0% (0) | 7.4% (4) | 0.0% (0) | 7.3% (4) | 0.0% (0) | 0.0% (0) | 10.0% (2) | 4.0% (2) | 0.0% (0) | 6.3% (4) | 0.0% (0) | 6.3% (3) | 2.0% (1) |
| MST (*Q*_1_, *Q*_3_)^b^ | 24 (14, 32) | 23 (12, 29) | 26 (15, 34) | 34 (18, 48) | 22 (12, 28) | 20 (15, 26) | 28 (2, 34) | 26 (18, 32) | 20 (18, 25) | 28 (12, 40) | 20 (11, 26) | 25 (16, 30) | 18 (2, 34) | 22 (15, 35) | 24 (10, 32) |
| EMST (SE)^b^ | 62 (3) | 66 (3) | 50 (6) | 73 (3) | 49 (5) | 69 (4) | 53 (6) | 53 (8) | 74 (5) | 63 (4) | 51 (7) | 67 (3) | 54 (6) | 55 (5) | 70 (4) |
| MFT (*Q*_1_, *Q*_3_)^b^ | 84 (29, 84) | 84 (34, 84) | 44 (23, 84) | 84 (60, 84) | 33 (18, 84) | 84 (52, 84) | 66 (28, 84) | 51 (25, 84) | 84 (72, 84) | 84 (34, 84) | 56 (20, 84) | 84 (32, 84) | 84 (18, 84) | 84 (56, 84) | 56 (25, 84) |
| TFT^b^ | 0~84 | 0~84 | 0~84 | 0~84 | 0~84 | 0~84 | 0~84 | 0~84 | 0~84 | 0~84 | 0~84 | 0~84 | 0~84 | 0~84 | 0~84 |
| ^a^Abbreviations: CSP, Cumulative survival probability (%); WR, Withdrawal rate (%); MST, Median survival time; EMST, Estimated means for survival time; SE, std error; MFT, Median follow-up time; TFT, Total follow-up time  ^b^month stated otherwise | | | | | | | | | | | | | | | |
