# Supplementary material for: Overexpression of proteasomal activator PA28α serves as a prognostic factor in oral squamous cell carcinoma
Source: J Exp Clin Cancer Res. 2016 Feb 19;35:35. doi: 10.1186/s13046-016-0309-z (PMC4759779; doi:10.1186/s13046-016-0309-z)
Supplement: Additional file 6: Figure S3. — The curves showed that the survival in the subtypes divided by T-stage (A), Smoking (B) and Chemotherapy (C) and Lymphatic metastasis (D) were well separated. *P < 0.05, P Values were calculated by the log-rank test. (PPT 232 kb) [file 13046_2016_309_MOESM6_ESM.ppt]

## Slide 1
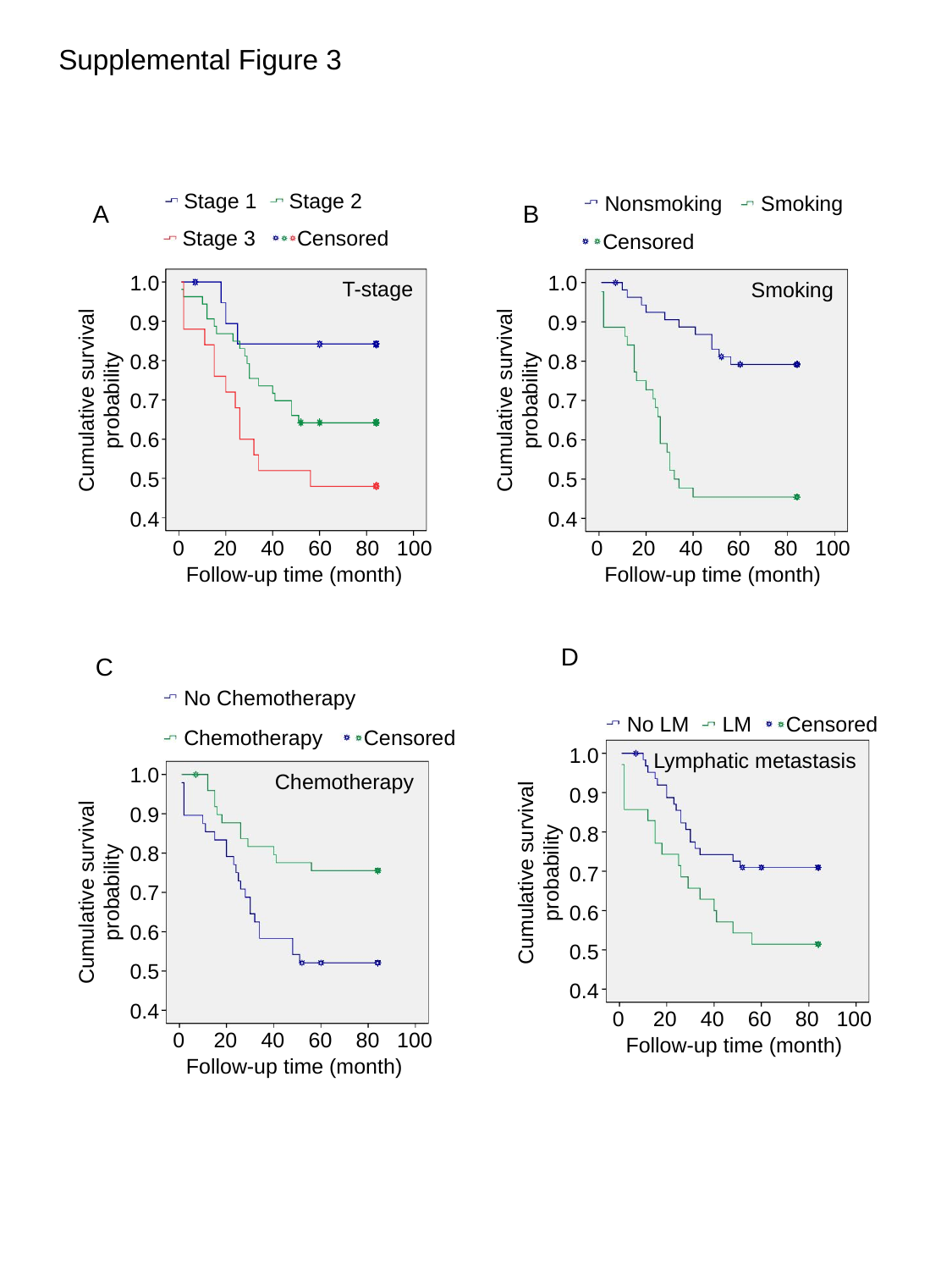

Supplemental Figure 3
Stage 1
Stage 2
Stage 3
Censored
T-stage
1.0
0.9
0.8
0.7
0.6
0.5
0.4
Cumulative survival probability
0
20
40
60
80
100
Follow-up time (month)
Nonsmoking
Smoking
Censored
Smoking
1.0
0.9
0.8
0.7
0.6
0.5
0.4
Cumulative survival probability
0
20
40
60
80
100
Follow-up time (month)
A
B
D
C
No Chemotherapy
Chemotherapy
Censored
Chemotherapy
1.0
0.9
0.8
0.7
0.6
0.5
0.4
Cumulative survival probability
0
20
40
60
80
100
Follow-up time (month)
No LM
LM
Censored
1.0
0.9
0.8
0.7
0.6
0.5
0.4
Lymphatic metastasis
Cumulative survival probability
0
20
40
60
80
100
Follow-up time (month)
